# Non-Destructive Inspection of Impact Damage in Composite Aircraft Panels by Ultrasonic Guided Waves and Statistical Processing

**DOI:** 10.3390/ma10060616

**Published:** 2017-06-04

**Authors:** Margherita Capriotti, Hyungsuk E. Kim, Francesco Lanza di Scalea, Hyonny Kim

**Affiliations:** 1 NDE&SHM Laboratory, University of California, San Diego, 9500 Gilman Drive, La Jolla, CA 92093, USA; flanzadiscalea@ucsd.edu; 2 Advanced Composites and Aerospace Structures Laboratory, University of California, San Diego, 9500 Gilman Drive, La Jolla, CA 92093, USA; hsk027@eng.ucsd.edu (H.E.K.); hyonny@ucsd.edu (H.K.)

**Keywords:** aerospace structures, ultrasonic guided waves, impact detection, NDE

## Abstract

This paper discusses a non-destructive evaluation (NDE) technique for the detection of damage in composite aircraft structures following high energy wide area blunt impact (HEWABI) from ground service equipment (GSE), such as heavy cargo loaders and other heavy equipment. The test structures typically include skin, co-cured stringers, and C-frames that are bolt-connected onto the skin with shear ties. The inspection exploits the waveguide geometry of these structures by utilizing ultrasonic guided waves and a line scan approach. Both a contact prototype and a non-contact prototype were developed and tested on realistic test panels subjected to impact in the laboratory. The results are presented in terms of receiver operating characteristic curves that show excellent probability of detection with low false alarm rates for defects located in the panel skin and stringers.

## 1. Introduction

Non-destructive evaluation (NDE) of aircraft structures is a crucial process to ensure passenger safety. Current visual inspection and lifespan estimation of aircraft are not able to properly assess the health status of the structures, especially when damages are present at the interior level, and can compromise the integrity of the overall assembly.

Composite aircraft, in particular, are subjected to a wide variety of damages that are very difficult to avoid and to visually detect. High energy wide area blunt impact (HEWABI), for example due to ground service equipment (GSE) maneuvers, is very common during aircraft operation and can cause major damages to the structure that are often not visible from the outside [1]. Such impacts are characterized by forces of large magnitudes and long-time scales [2] and can severely affect the structural integrity of key components (e.g., damage to stringers and C-frames), some of which are internal, and thus they are challenging to access from a one-sided (external only) NDE test.

Hence, there exists a need for a NDE tool that can help establish the need for further inspection following a GSE impact or similar event. The technique must be able to easily and rapidly inspect the structure, accessing it only from the outside, and to detect defects at different levels of the assembly in a statistically reliable manner.

There are many NDE techniques commonly used in the aerospace industry for composites; among them, visual inspection is still one of the most widely used, due to its simplicity and low cost. The requirement, not always possible, of accessibility, and the need of visualizing sub-surface and internal damages pushed researchers to develop more sophisticated technologies exploiting a wide variety of physics principles, such as ultrasonics, infrared thermography, shearography, and radiography [3,4]. Each one of these methods has advantages and drawbacks, addressing specific problems encountered in the inspection of different composite parts and joints of aircrafts and in detecting the multiple categories of damage [4].

This paper describes a method that utilizes ultrasonic guided waves (UGWs), non-contact transducers, and statistical processing to achieve the above-mentioned goals. The need for rapid inspection of the structure points to UGWs as suitable candidates. Among the hundreds of examples of UGW inspection of composite plates, a few examples can be found in Refs. [5,6,7,8,9,10,11,12,13,14,15,16,17]. While, to our knowledge, most of them aimed at the detection of sub-surface flaws (skin damage) and/or restricted the development of the technique to a laboratory environment, we focus on the detection of impact damage, that highly compromises the integrity of the aircraft [18], and we propose a field-applicable method. To do so, non-contact air-coupled transducers are employed. Their potential has been understood in the past [3] and tested [19,20], and is here used for damage detection on a wide aerospace specimen for the first time.

## 2. Test Panels

**Figure 1:**
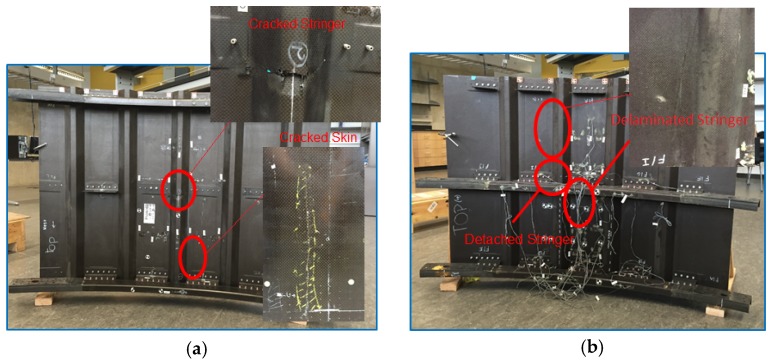

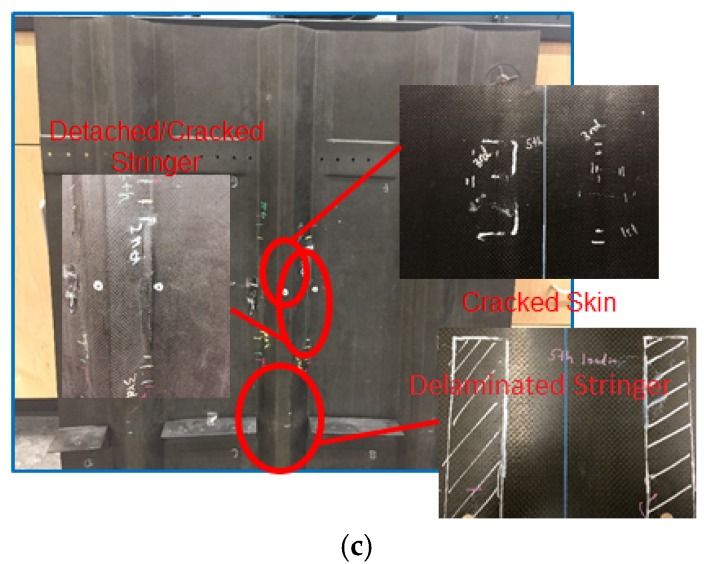
Test specimens: (**a**) Panel 1: five stringers, three C-frame panels with cracked skin, and a cracked stringer; (**b**) Panel 2: four stringers, three C-frame panels with a disbonded/detached stringer; (**c**) Panel 3: three stringers, two C-frame panels with cracked skin, a detached/cracked stringer, and a disbonded stringer.

For this study, carbon/epoxy composite panels representative of commercial aircraft construction (e.g., wide-body fuselage), manufactured and tested at the UCSD from a previous FAA-sponsored HEWABI project, [2], were used to develop and test the NDE technique. The panels were designed and manufactured following industry procedures and dedicated fabrication processes to achieve comparable aerospace characteristics and quality. While the test specimens varied in size, they were manufactured with the same material and overall structural design. Each test panel (Figure 1) was composed of a curved skin, stiffened by co-cured stringers along the longitudinal direction, and by C-frames (bolted to the skin by shear ties) along the radial direction. The lay-up of the skin was as shown in Table 1. All parts were cured via the autoclave process.

These panels were subjected to wide area blunt impacts by using a rubber impactor driven by a one degree of freedom actuator table to simulate a GSE in-service contact and resulting damage formation. Different damage types and severities at various locations were generated and surveyed by visual inspection and hand-held ultrasonic scanning. Detected damage included, from the outside skin into the C-frames: skin cracks, stringer–skin disbonds or detachments, stringer cracks, shear tie cracks, and C-frame cracks. Some of these are highlighted in Figure 1.

## 3. Ultrasonic Guided Wave Inspection Concept

**Figure 2:**
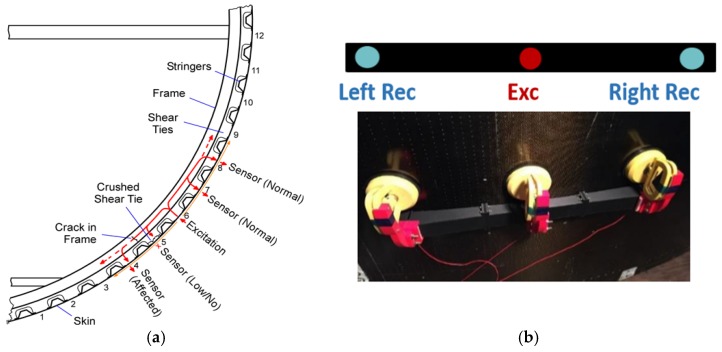
(**a**) Schematic of the ultrasonic guided waves (UGW) approach for the aerospace panel inspection; (**b**) differential scheme for contact inspection.

The nature of the structure suggested the use of UGW: the ultrasonic waves can travel inside the different components of the assembly that behave as natural waveguides. Moreover, the complexity of the panels, in terms of size, curvature, material, and number of interfaces and travelling paths, requires wide area coverage and low attenuation, properties that can be satisfied by UGW. The schematic below (Figure 2a) shows the adopted inspection approach: the excitation is sent from the outside skin, the wave penetrates into the stringers and shear ties, travels into the C-frames and, after interacting with defects if present, can be collected again from receivers placed on the outer skin.

**Figure 3:**
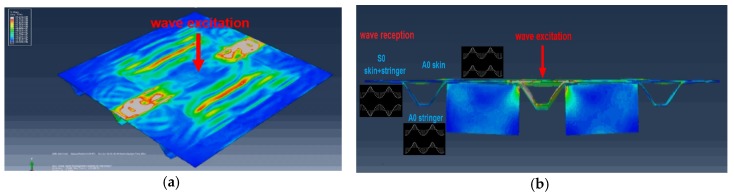
FE model of a stiffened composite panel: (**a**) 3D view; (**b**) cross-sectional view showing multi-mode wave propagation and acoustic inter-talk between the skin, stringer, and shear ties.

Numerical Finite Element (FE) studies of UGW propagation in this kind of stiffened panel were previously conducted in [17] in the context of real-time impact force identification. Those simulations also supported the idea of exploiting the multiple wave modes simultaneously present in this structure. Figure 3 shows some results from FE simulations conducted using the procedure detailed in [17], where it is possible to notice the presence of the two fundamental modes (anti-symmetric A_0_ and symmetric S_0_) and their propagation into the different components. The specific excitation used in this figure was an impulsive force of 0.1 ms duration, therefore generating a usable frequency bandwidth up to 10 kHz. These kinds of simulations aided the time gating of the various wave modes measured in the experimental tests that are discussed in the next sections.

## 4. Statistical Processing

**Figure 4:**
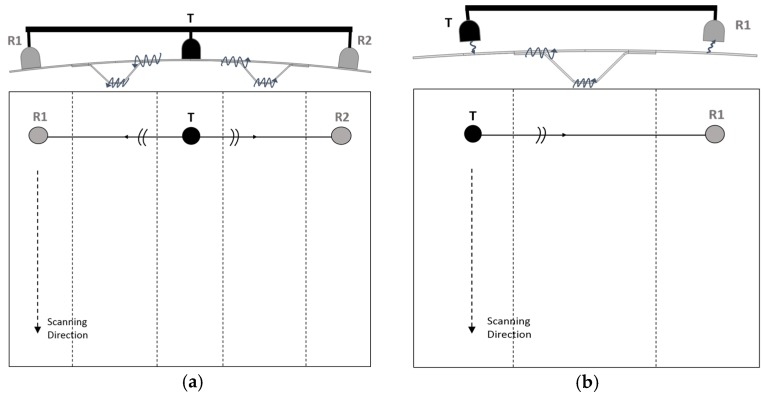
Line scanning approach for (**a**) a contact system and (**b**) a non-contact system; cross-sectional view (top drawings) and front view (bottom drawings).

Due to the requirement of rapid inspection, a line scanning approach of UGW testing was taken. Accordingly, the test panels were scanned along a stringer direction, as shown in Figure 4.

This scanning process also lends itself to a statistical analysis aimed at minimizing signal behavior due to normal operational variability across a scan (specimen inhomogeneities, etc.) and maximizing signal variations due to true structural defects. Following the general statistical Multivariate Outlier Analysis (MOA) for novelty detection [21,22], the test scans were normalized by their normal statistical distribution (“baseline”). A baseline is built relying on signals collected from a known pristine area of the impacted specimen; every test scan is then compared to its baseline through a MOA processor. Relying on the physics of wave propagation, the latter extracts specific features from each UGW signal and feeds them into a feature vector. A damage index (*DI*) is computed according to the Mahalanobis squared distance metric:

(1) $$DI=\left(x-\bar{x}\right)\;\times \;C^{-1}\;\times \;{\left(x-\bar{x}\right)}^{T}$$

where x is the feature vector, $\bar{x}$ is the baseline average vector, and C is the baseline covariance matrix. A large value of *DI* represents a deviation from the normal statistics of the signals, hence is an outlier in the distribution or, in a damage detection perspective, a defect.

The feature extraction process is performed on selected time-gated wave packets, corresponding to different wave modes propagating in the stiffened panel structure. This selection relies on velocity information about the various propagating modes and enhances the sensitivity of the technique to specific defects.

## 5. Experimental Implementation

### 5.1. Contact Technique

The initial development of the NDE technique used contact PZT transducers to excite and detect the UGWs in the test panels. Conventional ultrasonic gel couplant was used. Moreover, the contact approach utilized a differential detection scheme that relies on the “imbalance” of the signal received on two opposite sides of the transmitter to detect a possible defect. The contact prototype with differential scheme is shown in Figure 2b. The differential scheme, which the UCSD has used effectively in another NDE project that required scanning across a test structure [23], is robust against coupling variations of the transmitter and several other changes not associated to damage. A narrowband PZT transmitter centered at 150 kHz was used in conjunction with two receivers centered at the same frequency (R15S, Mistras, Princeton Jct, NJ, USA). A five-cycle toneburst with Hanning modulation at 150 kHz was used as the excitation signal. A National Instruments PXI (National Instruments, Austin, TX, USA) unit running under LabVIEW was used as the signal generation and acquisition instrument. At each scan line, the UGWs were collected by the two receivers, gated in time, and processed to extract features related to the imbalance between the two (e.g., ratios of amplitudes). The specific features used are listed in Table 2 ($x_{1/2}$ refers to the signal from receiver 1 and 2, respectively):

These features were then fed into the feature vector and used to compute the *DI* metric according to Equation (1). The scan resolution across the damaged areas was approximately 1 cm. 

**Figure 5:**
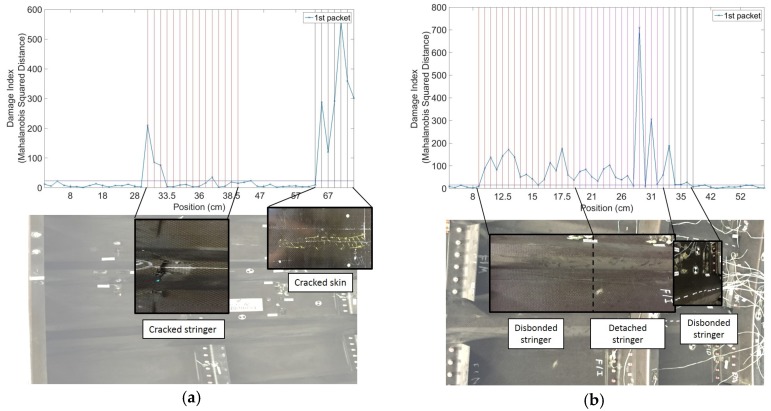
Representative results from contact UGW scans: (**a**) Panel 1; (**b**) Panel 2.

Representative results are shown in Figure 5a,b for Panel 1 and Panel 2, respectively. The vertical lines represent known positions of defects from the prior visual surveys and ultrasonic scans. It can be seen how the *DI* increases noticeably in known damaged areas, with very low noise levels, owing to the statistical outlier analysis. As expected from a skin-probing technique, the sensitivity to damage is higher for the skin defects than for the stringer defects, although the latter are also clearly visible over the very low noise floor of the pristine structure.

**Figure 6:**
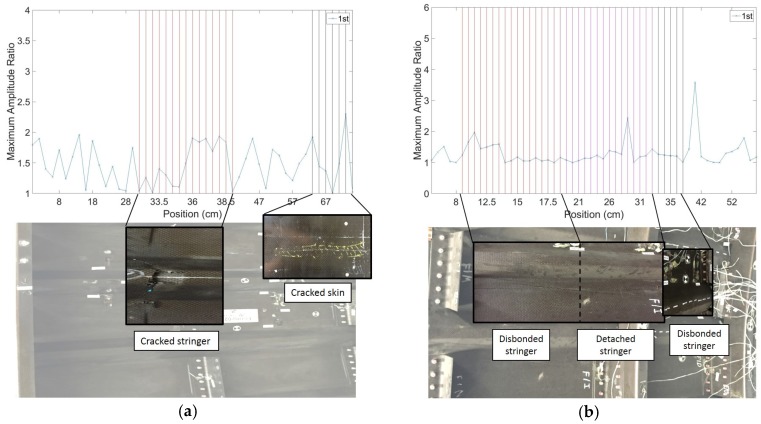
Amplitude ratio from contact UGW scans: (**a**) Panel 1; (**b**) Panel 2.

Using a traditional B-scan that simply relies on the maximum amplitude of the signal with no statistical processing, no reliable detection of damage could be achieved. As shown in Figure 6a,b, the ratio between the maximum amplitude of the signals collected at receivers 1 and 2 is a highly variable metric, with poor robustness of defect detection. The statistical analysis improves the reliability of the result, since it normalizes the metric by the “normal” statistics of the structure.

These promising results led to the development of the next-generation prototype that does not require contact with the test structure as described in the next section. 

### 5.2. Non-Contact Technique

**Figure 7:**
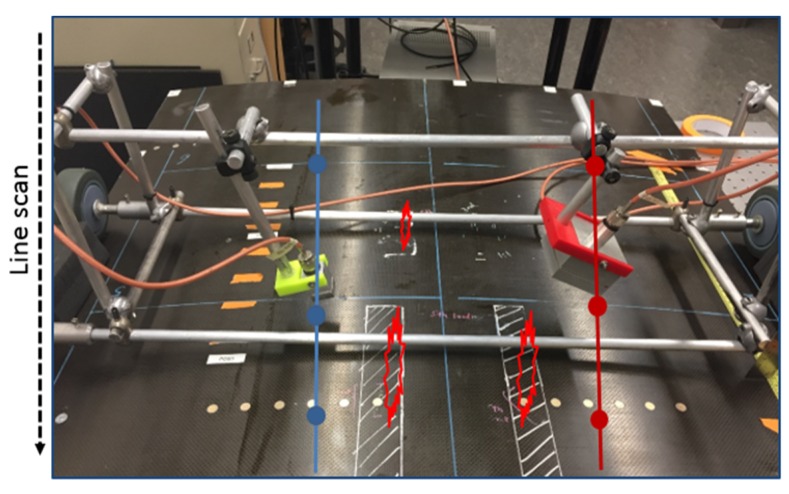
The non-contact air-coupled scanning prototype mounted on Test Panel 3.

To ease the applicability of a rapid scanning technique for an actual field application, a non-contact version of the UGW system was designed, constructed, and tested which omits the need of couplant application per scan. Shown in Figure 7, the non-contact scanning prototype consists of a cylindrically focused air-coupled transmitter (NCG200-S50-C76-EP-X, Ultran, Hoboken, NJ, USA) (right-hand side in Figure 6) and an unfocused air-coupled receiver (NCG200-S19, Ultran, Hoboken, NJ, USA) operated in a pitch–catch mode. The transducers (both narrowband with a central frequency of 170 kHz) are mounted on a moving cart that allows the rapid and consistent scanning of the test structure. The stability of the non-contact coupling removes the need for the differential approach, such that a simple pitch–catch test scheme is appropriate. The transducers are also oriented at angles that maximize the transduction of the dominant flexural mode in the panel’s skin [10,24]. Both transducers are piezocomposite devices that minimize the acoustic impedance mismatch with air for maximum ultrasonic signal transmission and reception. The excitation signal is a five-cycle toneburst with Hanning modulation centered at 170 kHz. The same NI PXI unit used in the contact setup was used for signal excitation and acquisition.

**Figure 8:**
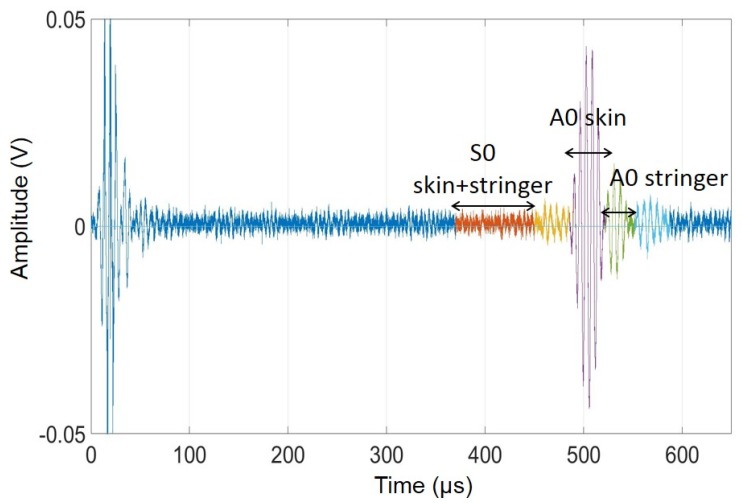
Typical RF waveform measured by the air-coupled pitch–catch prototype from Test Panel 3.

The previously mentioned multi-mode wave propagation was exploited in the non-contact technique. A typical received RF (Radio Frequency) waveform from Test Panel 3 is shown in Figure 8. It is possible to notice different arrivals. Measurements from contact PZT transducers mounted at specific locations of the panel, as well as group velocity information from the FE analysis, allowed to separate the dominant flexural mode traveling primarily in the panel skin from that leaking from the skin into the co-cured stringers.

The multivariate outlier analysis allows to select individual modes (from time gating) to build the feature vector. The features chosen are related to the signal strength and velocity and are listed in Table 3, where $x_{p}$ refers to the signal and *p* identifies each wave mode packet used.

**Figure 9:**
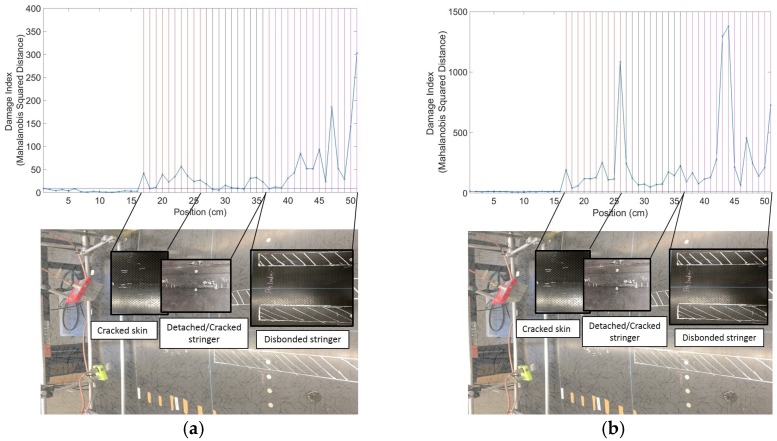
Representative results from non-contact (air-coupled) UGW scans of Panel 3: (**a**) skin modes only; (**b**) skin modes plus stringer modes.

Figure 9 presents the *DI* trace obtained from the line scan of Test Panel 3 using (a) only skin modes, and (b) skin and stringer modes. It can be noticed how adding the stringer modes results in an enhanced defect detection sensitivity, especially for the stringer defects.

**Figure 10:**
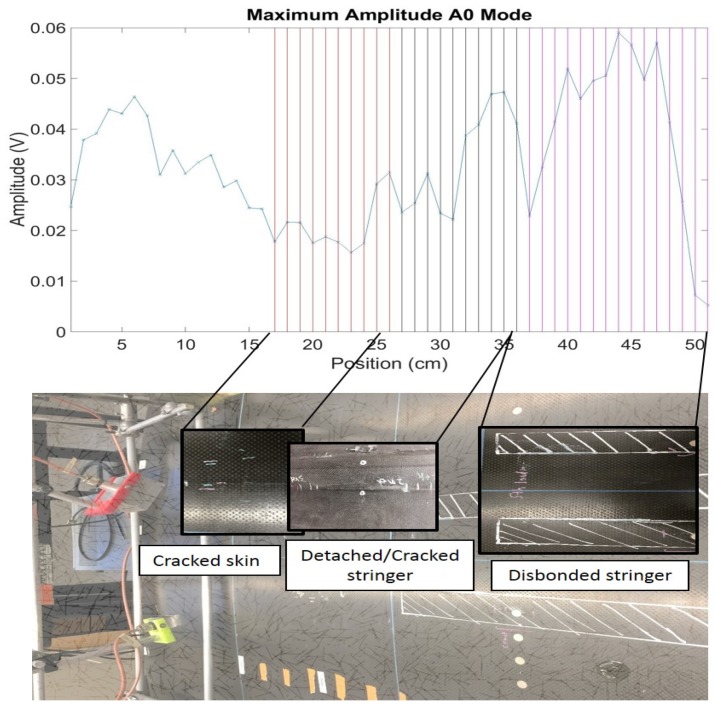
Maximum amplitude from non-contact UGW scans: Panel 3.

Again, the B-scan for the maximum amplitude value is reported in Figure 10, to show the benefit of the statistical analysis.

## 6. Receiver Operating Characteristic Curves

In order to properly assess the performance of the tests, receiver operating characteristic (ROC) curves were computed [25]. These plots compare the probability of detection (POD) to the probability of false alarm (PFA) for different threshold levels applied to the *DI* traces.

ROC curves were computed for each defect type at varying *DI* thresholds: a curve located in the upper left corner of the plot indicates a good defection performance (high POD and low PFA). The area under the curve (AUC) is a metric that summarizes this goodness in performance. The dashed straight line represents the 50–50 guess.

**Figure 11:**
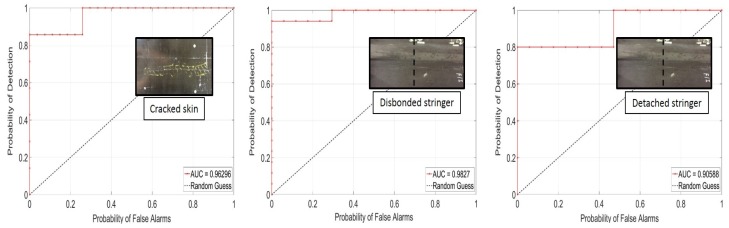
Receiver operating characteristic (ROC) curves for the contact non-destructive evaluation (NDE) technique: cracked skin, disbonded stringer, and detached stringer defects (Panel 1 and Panel 2).

Figure 11 shows the ROC curves for the contact NDE prototype on Test Panel 1 and Test Panel 2 for the disbonded stringer, the detached stringer, and the cracked skin types of defects. Each symbol in the curves represents a threshold level applied to the *DI* traces from the line scans. These results indicate, for example, that the cracked skin defect can be detected with an 86% POD and a 0% PFA or, alternatively, with a 100% POD and 26% PFA. Similarly, the disbonded stringer defect can be detected with a 94% POD and 0% PFA or, alternatively, with a 100% POD and 29% PFA. A somewhat worse performance was found for the detached stringer defect, where an 80% POD affords a 0% PFA (and a 100% POD results in a 47% PFA).

**Figure 12:**
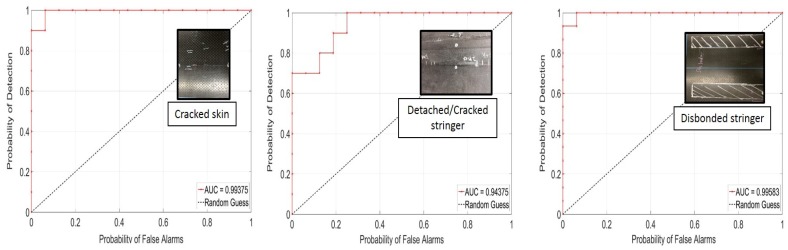
ROC curves for the non-contact NDE technique (skin modes only): cracked skin, detached stringer, and disbonded stringer defects (Panel 3).

**Figure 13:**
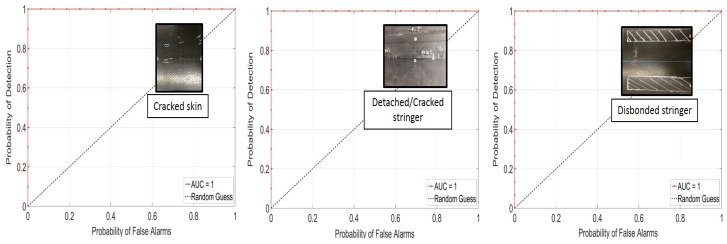
ROC curves for the non-contact NDE technique (skin and stringer modes): cracked skin, detached stringer, and disbonded stringer defects (Panel 3).

Figure 12 and Figure 13 show the ROC curves for the non-contact NDE technique applied to Test Panel 3. Figure 10 shows the results obtained using the skin modes only. The detection performance is excellent, especially considering the non-contact nature of the coupling. The best detection performance was found for the cracked skin and the disbonded stringer defect (e.g., 100% POD with less than 10% PFA), with a somewhat worse performance for the detached/cracked stringer defect (e.g., 100% POD with 25% PFA, or 70% POD with 0% PFA).

In Figure 13, the stringer modes are added to the skin modes in the feature vector. This combination results in a perfect detection performance for all defects considered, i.e., a 100% POD with 0% PFA. The reason for this improvement is that the acoustic cross-talk between the skin and stringer is such that modes primarily propagating into one of the two components are sensitive to defects in the other component, in addition to defects in the same component.

## 7. Conclusions

This paper summarizes a rapid NDE technique for the detection of structural defects in composite aircraft structures subjected to ground service equipment (GSE) impacts. The approach taken utilizes the waveguide geometry of the structure by means of ultrasonic guided waves. Two laboratory prototypes for line scanning were developed, one using contact PZT transducers with a differential approach and one using non-contact (air-coupled) transducers in a pitch–catch approach. The inspection utilizes a statistical outlier analysis that compensates each measurement for the normal (baseline) variation during a scan, thereby increasing the POD (true detections) and decreasing the PFA (false positive). Tests conducted on previously impacted test panels representative of commercial aircraft construction indicated an excellent detection performance (in terms of POD vs. PFA tradeoffs) for skin and stringer defects. A perfect detection for these defects was actually obtained by the non-contact system once both skin wave modes and stringer wave modes were combined in the statistical feature vector.

The prototypes discussed here are early-stage laboratory systems that do not include automatic data processing of the scan. Ongoing work is aimed at adding a tachometer to track the position of the inspection head and implementing automatic signal processing to generate the scan output in quasi-real time.

The defects tested in this study were limited to the panel skin and stringers. Defects located in the deeper structure, specifically shear ties and C-frames, were not targeted, because the frequencies used were found to be too high to penetrate these regions. Ongoing work is aimed at testing lower UGW frequencies in an effort to penetrate into the C-frames to provide comprehensive coverage of the structure.

## Figures and Tables

**Table 1:** Composite Panel Skin Lay-Up.

Ply #	Material	Ply Thickness (mm)	Orientation Angle (°)
1	Plain weave fabric	0.208	0
2–17	Unidirectional	0.14	[0/45/90/−45/0/45/90/−45]_S_
18	Plain weave fabric	0.208	0

**Table 2:** Contact Technique Features List.

Feature #	Feature Name	Feature Extraction
1	RMS ratio	$\frac{RMS\left(x_{1}\right)}{RMS\left(x_{2}\right)}$
2	Maximum value ratio	$Max\left(\frac{Max\left|x_{1}\right|}{Max\left|x_{2}\right|},\frac{Max\left|x_{2}\right|}{Max\left|x_{1}\right|}\right)$
3	Area under packet ratio	$\frac{Area\left(x_{1}\right)}{Area\left(x_{2}\right)}$
4	Peak to peak normalized difference	$\frac{\left|Ppk\left(x_{1}\right)-Ppk\left(x_{2}\right)\right|}{\sqrt{Ppk\left(x_{1}\right)\;\times \;Ppk\left(x_{2}\right)}}$
5	Area under FFT normalized difference	$\frac{\left|AreaFFT\left(x_{1}\right)-AreaFFT\left(x_{2}\right)\right|}{\sqrt{AreaFFT\left(x_{1}\right)\;\times \;AreaFFT\left(x_{2}\right)}}$
6	Maximum value cross-correlation normalized difference	$\frac{\mathrm{Max}\left(xcorr\left(x_{1},x_{2}\right)\right)}{\sqrt{\mathrm{Max}\left(xcorr\left(x_{1}\right)\right)\;\times \;\mathrm{Max}\left(xcorr\left(x_{2}\right)\right)}}$
7	Variance normalized difference	$\frac{\left|Var\left(x_{1}\right)-Var\left(x_{2}\right)\right|}{\sqrt{Var\left(x_{1}\right)\;\times \;Var\left(x_{2}\right)}}$
8	RMS normalized difference	$\frac{\left|RMS\left(x_{1}\right)-RMS\left(x_{2}\right)\right|}{\sqrt{RMS\left(x_{1}\right)\;\times \;RMS\left(x_{2}\right)}}$

where RMS is the Root Mean Square value and FFT stands for Fast Fourier Transform.

**Table 3:** Non-Contact Technique Features List

Feature #	Feature Name	Feature Extraction
1	Maximum value	$Max\left|x_{p}\right|$
2	Index of maximum value	$Ind\left(Max\left|x_{p}\right|\right)$
3	Variance	$Var\left(x_{p}\right)$
4	Kurtosis	$Kurt\left(x_{p}\right)$

## Supplementary Materials

Supplementary File 1
