# Supplementary figures and images for: Non-Destructive Inspection of Impact Damage in Composite Aircraft Panels by Ultrasonic Guided Waves and Statistical Processing
Source: Materials (Basel). 2017 Jun 4;10(6):616. doi: 10.3390/ma10060616 (PMC5553524; doi:10.3390/ma10060616)

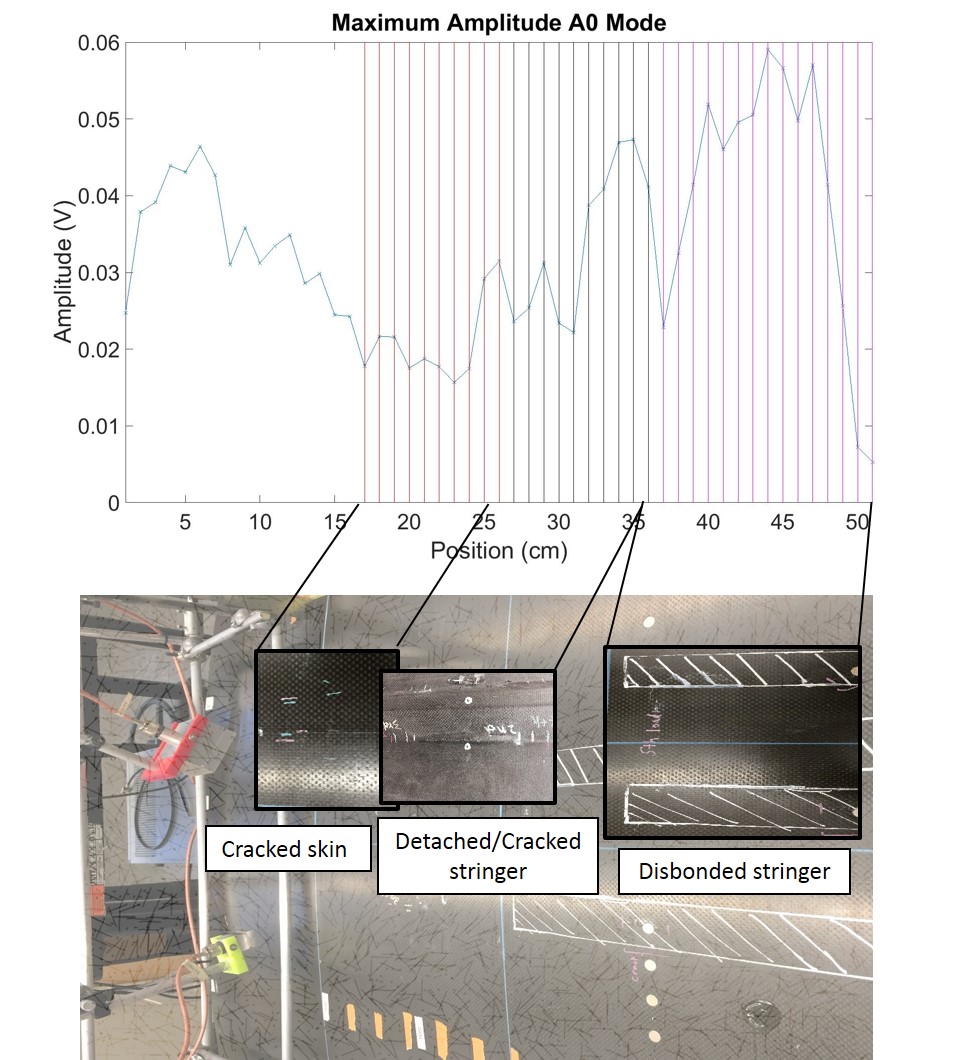

Supplement: Supplementary File 1 [file materials-10-00616-s001.zip › Figures_rev/Figure S10_Maximum Amplitude of A0 Mode from noncontact (air-coupled) UGW scans of Panel 3.jpg]

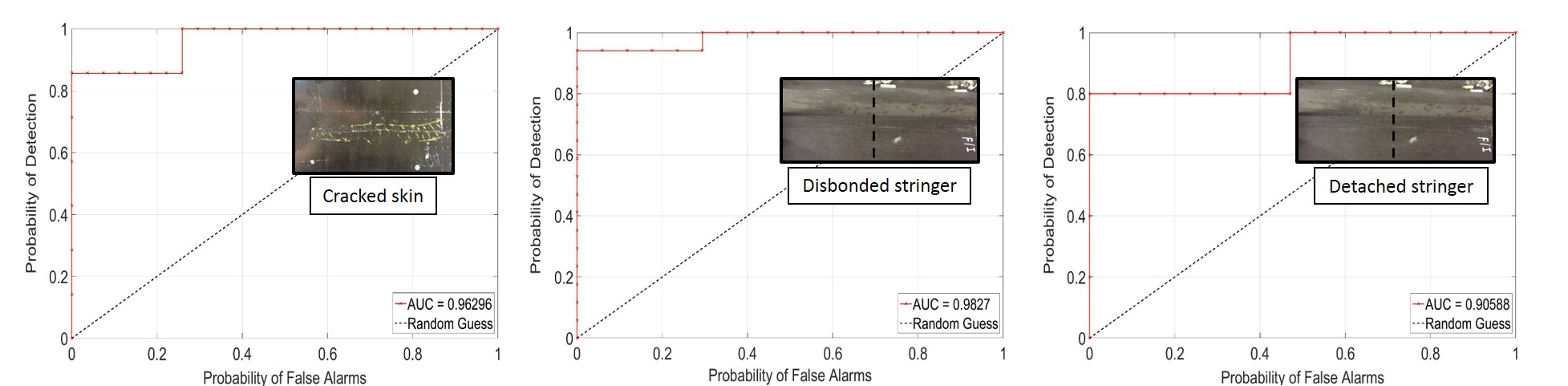

Supplement: Supplementary File 1 [file materials-10-00616-s001.zip › Figures_rev/Figure S11_ROC curves for the contact NDE technique (Panel 1 and Panel 2).jpg]

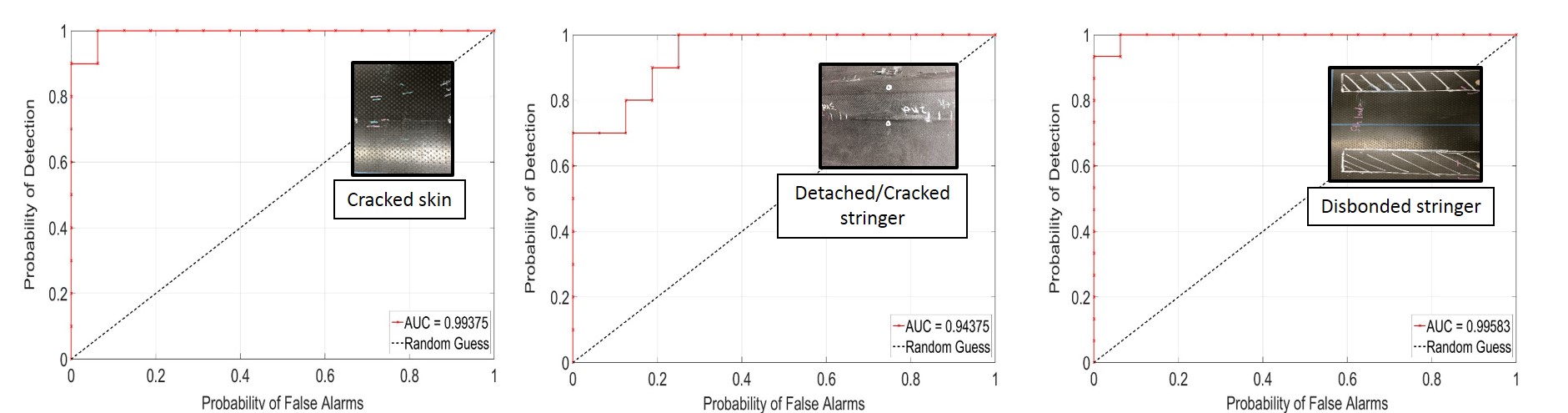

Supplement: Supplementary File 1 [file materials-10-00616-s001.zip › Figures_rev/Figure S12_ROC curves for the non contact NDE technique skin modes only (Panel 3).jpg]

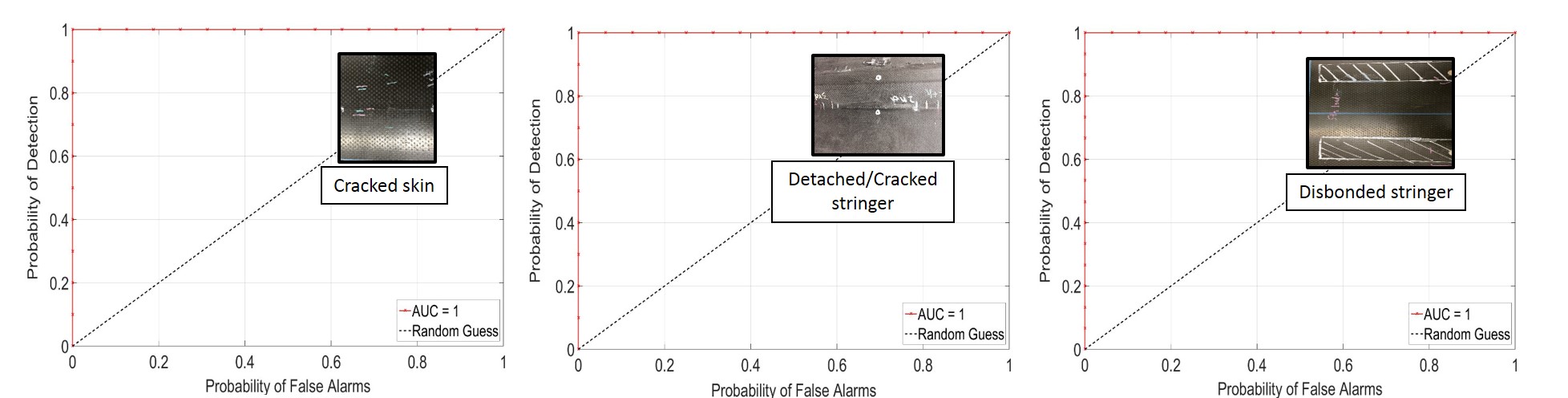

Supplement: Supplementary File 1 [file materials-10-00616-s001.zip › Figures_rev/Figure S13_ROC curves for the non contact NDE technique skin and stringer modes (Panel 3).jpg]

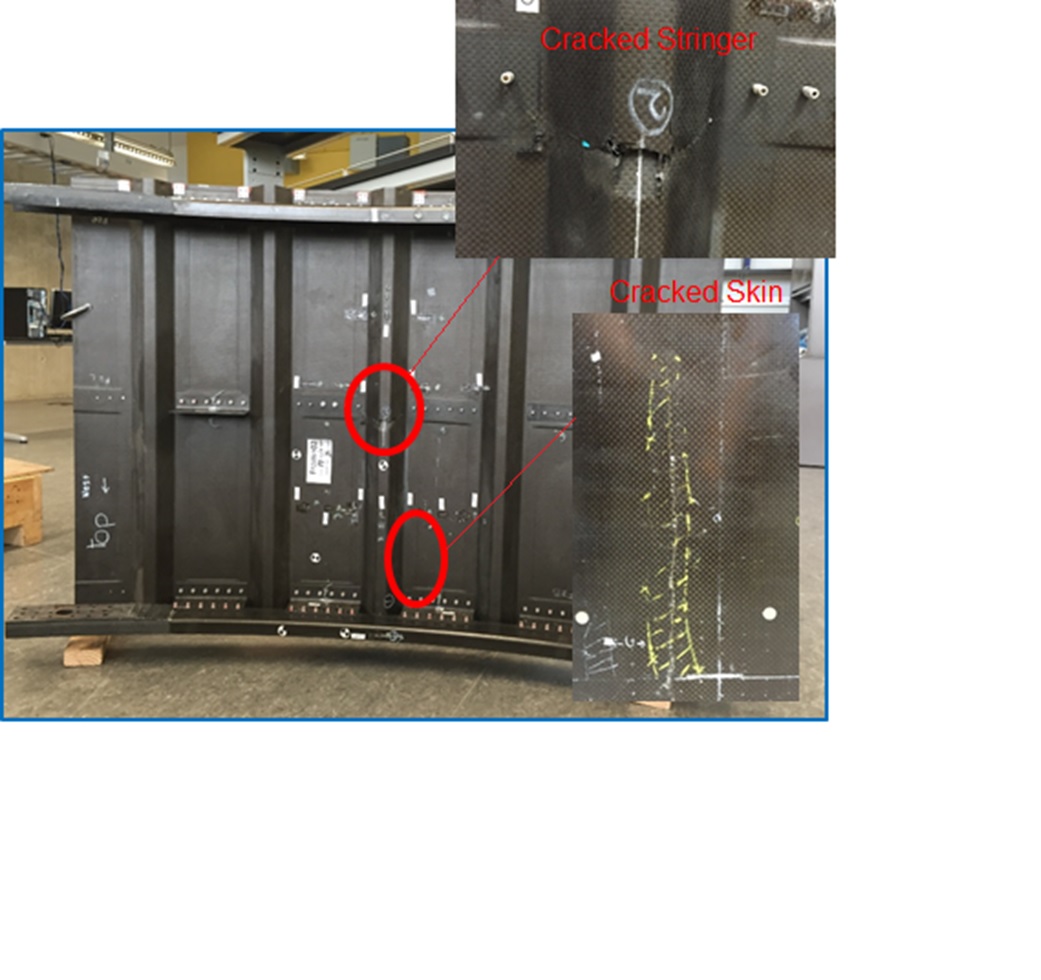

Supplement: Supplementary File 1 [file materials-10-00616-s001.zip › Figures_rev/Figure S1a_Test specimens (a)Panel 1.jpg]

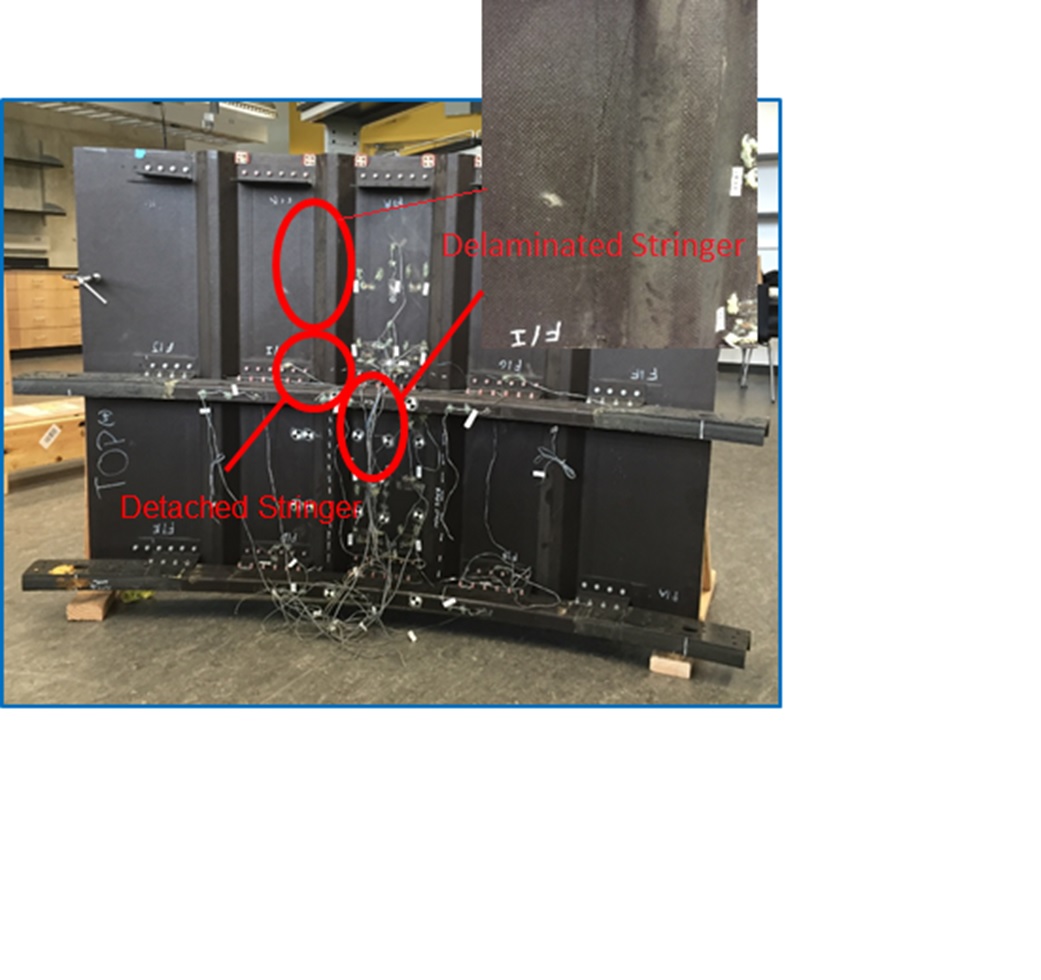

Supplement: Supplementary File 1 [file materials-10-00616-s001.zip › Figures_rev/Figure S1b_Test specimens (b)Panel 2.jpg]

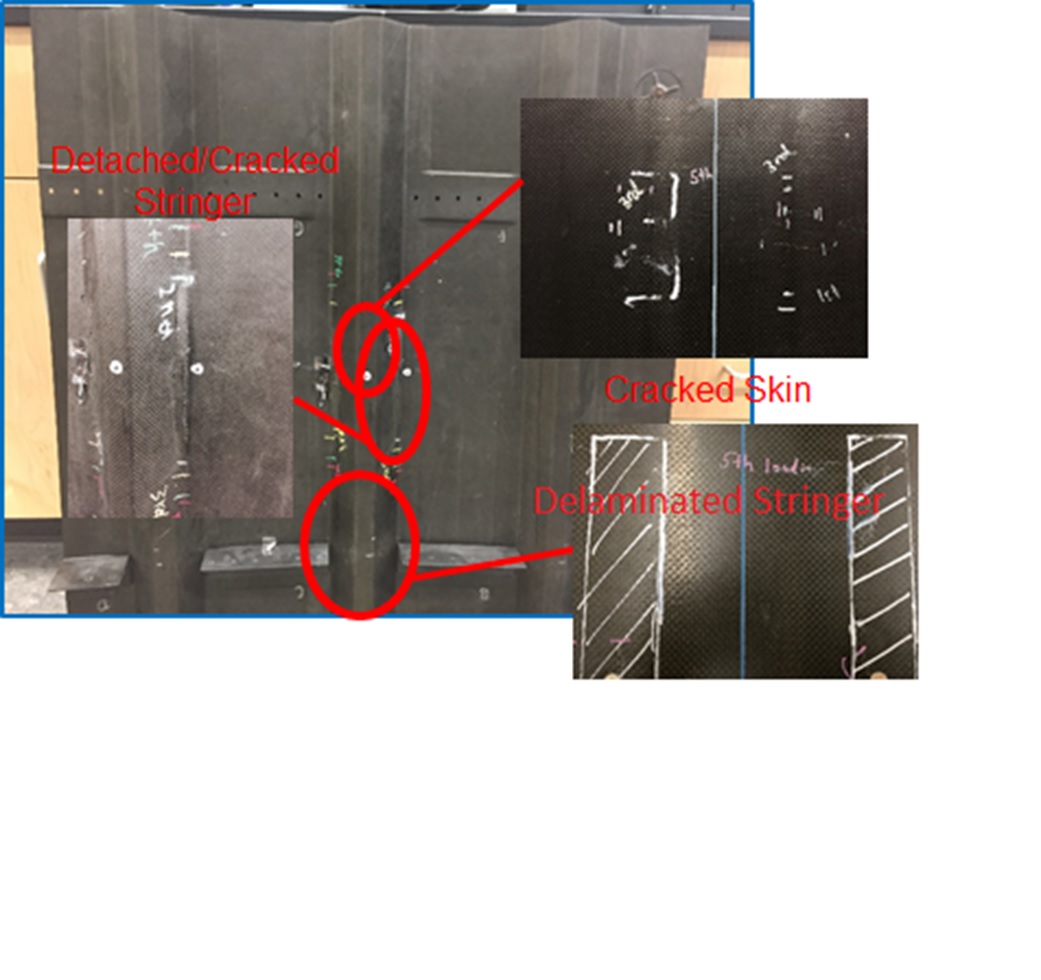

Supplement: Supplementary File 1 [file materials-10-00616-s001.zip › Figures_rev/Figure S1c_Test specimens (c)Panel 3.jpg]

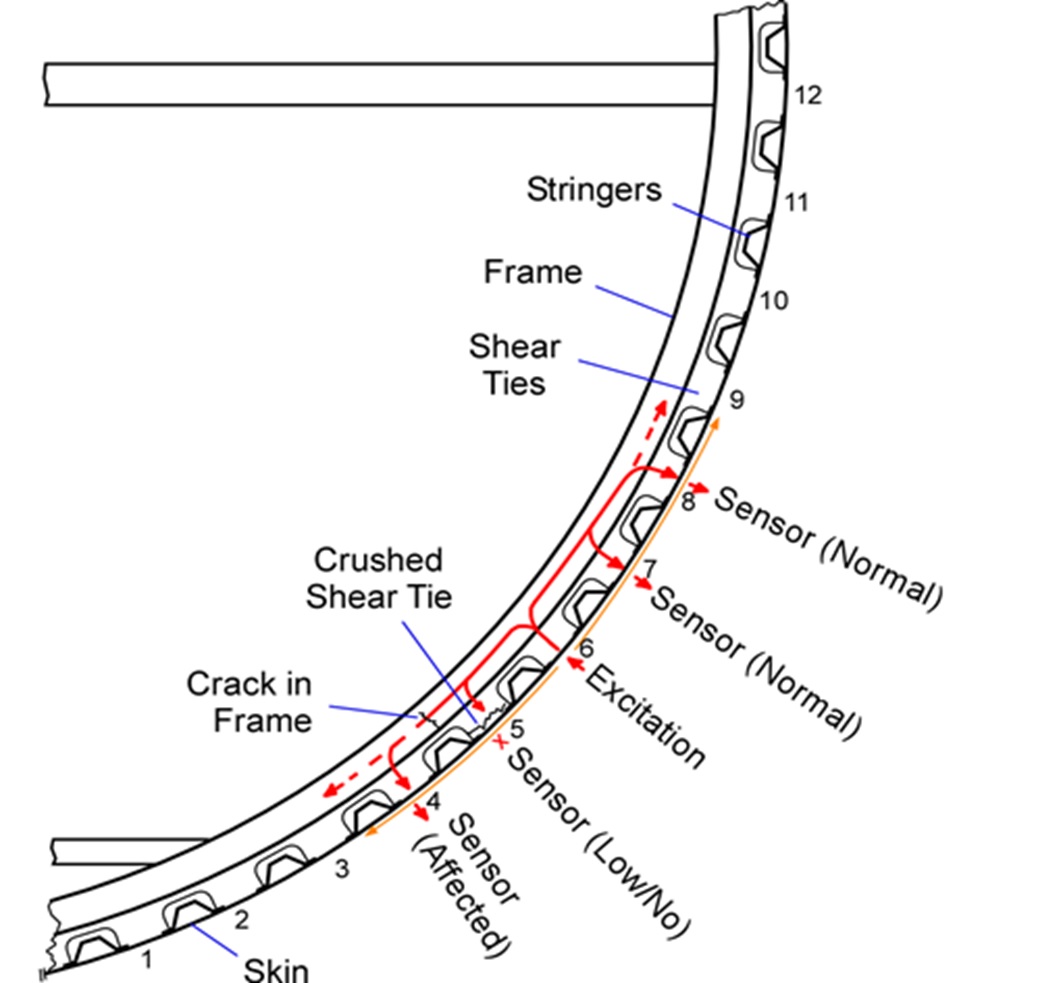

Supplement: Supplementary File 1 [file materials-10-00616-s001.zip › Figures_rev/Figure S2a_Schematic of the UGW approach for the aerospace panel inspection.jpg]

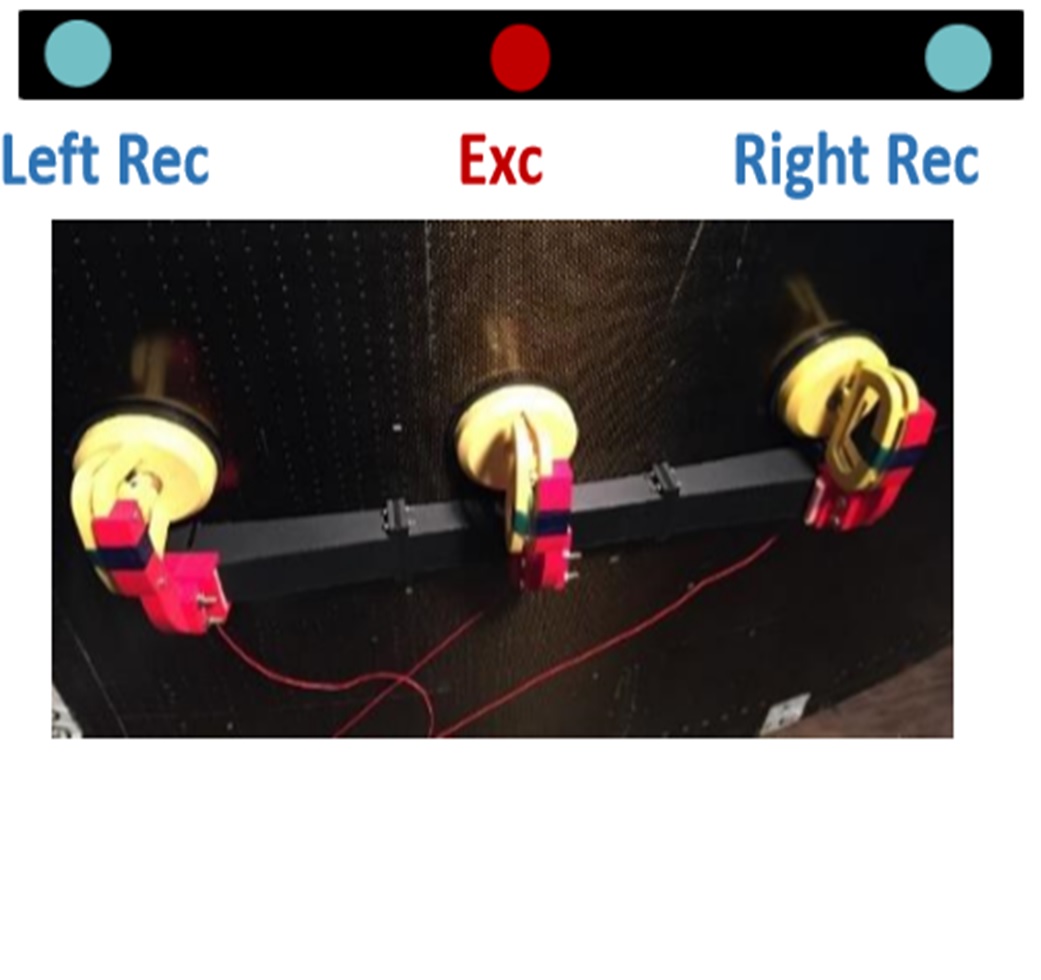

Supplement: Supplementary File 1 [file materials-10-00616-s001.zip › Figures_rev/Figure S2b_Differential scheme for contact inspection.jpg]

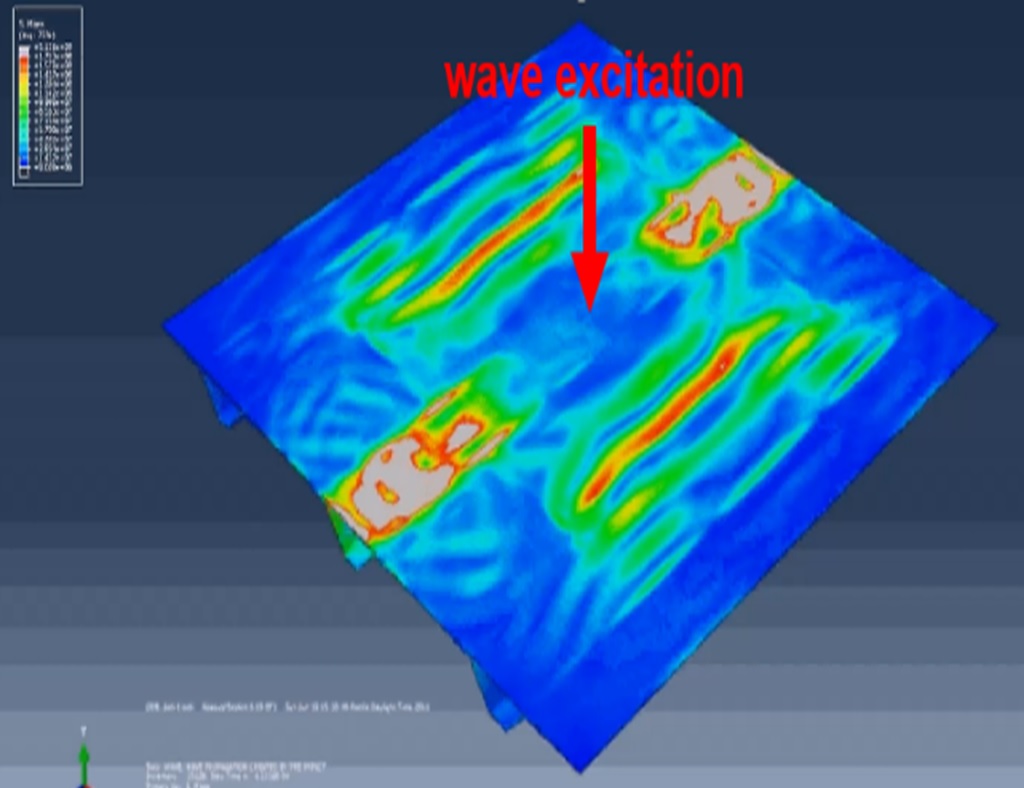

Supplement: Supplementary File 1 [file materials-10-00616-s001.zip › Figures_rev/Figure S3a_FE model of a stiffened composite panel (a) 3D view.jpg]

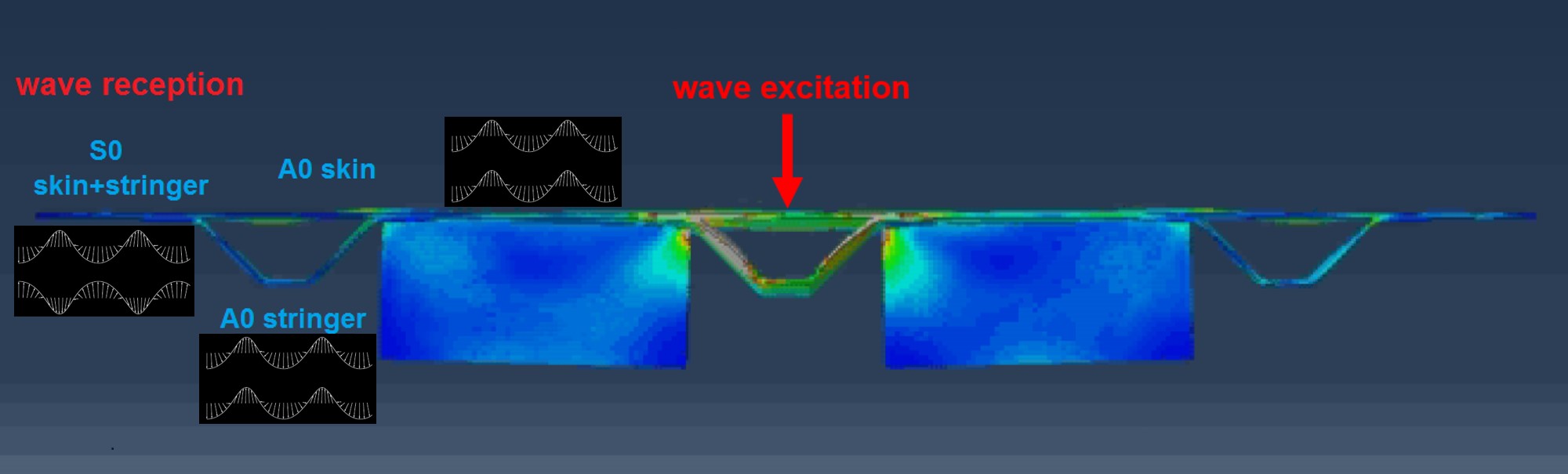

Supplement: Supplementary File 1 [file materials-10-00616-s001.zip › Figures_rev/Figure S3b_FE model of a stiffened composite panel (b) cross-sectional view_rev.jpg]

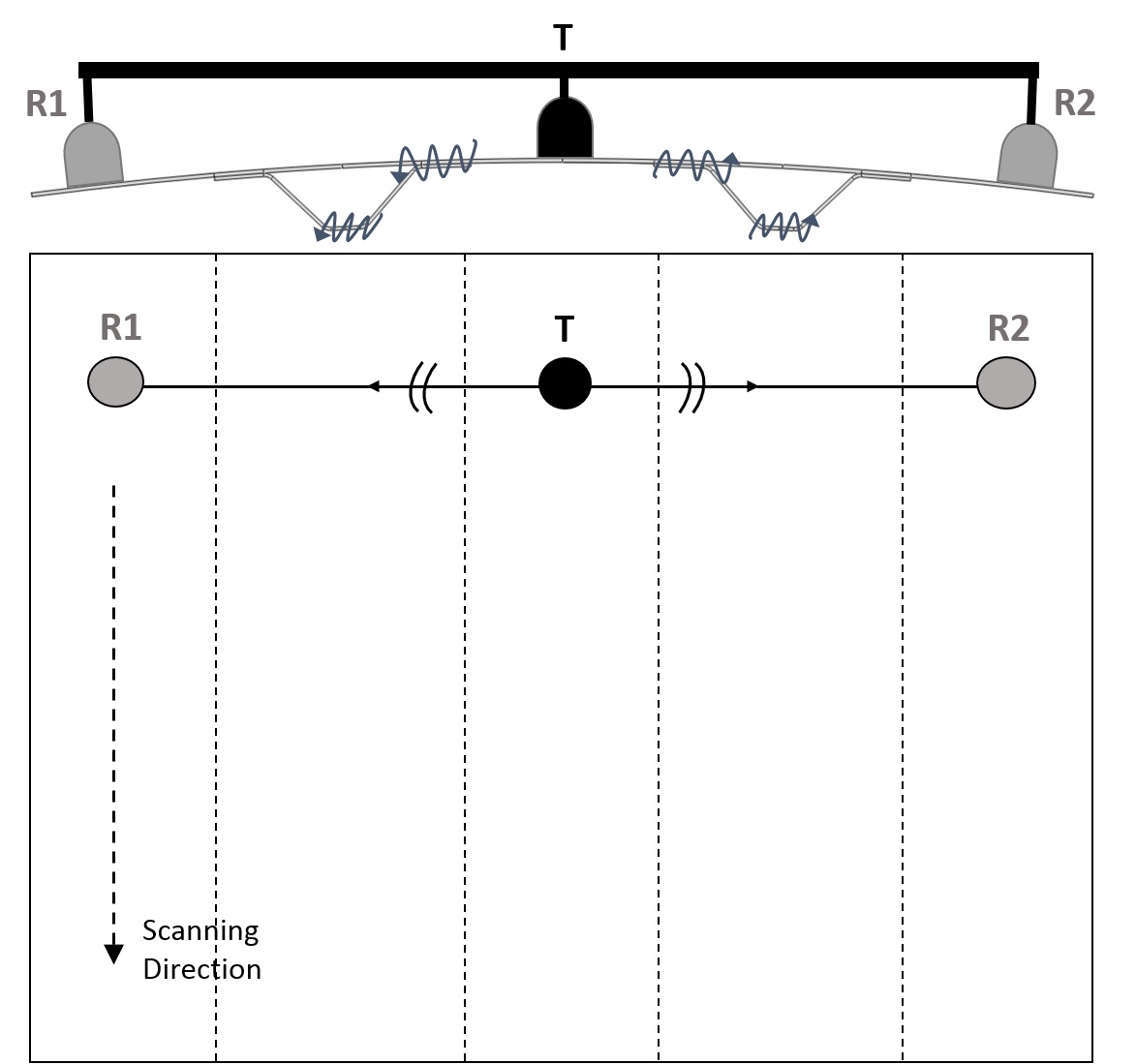

Supplement: Supplementary File 1 [file materials-10-00616-s001.zip › Figures_rev/Figure S4a_Line scanning approach for (a) contact system.jpg]

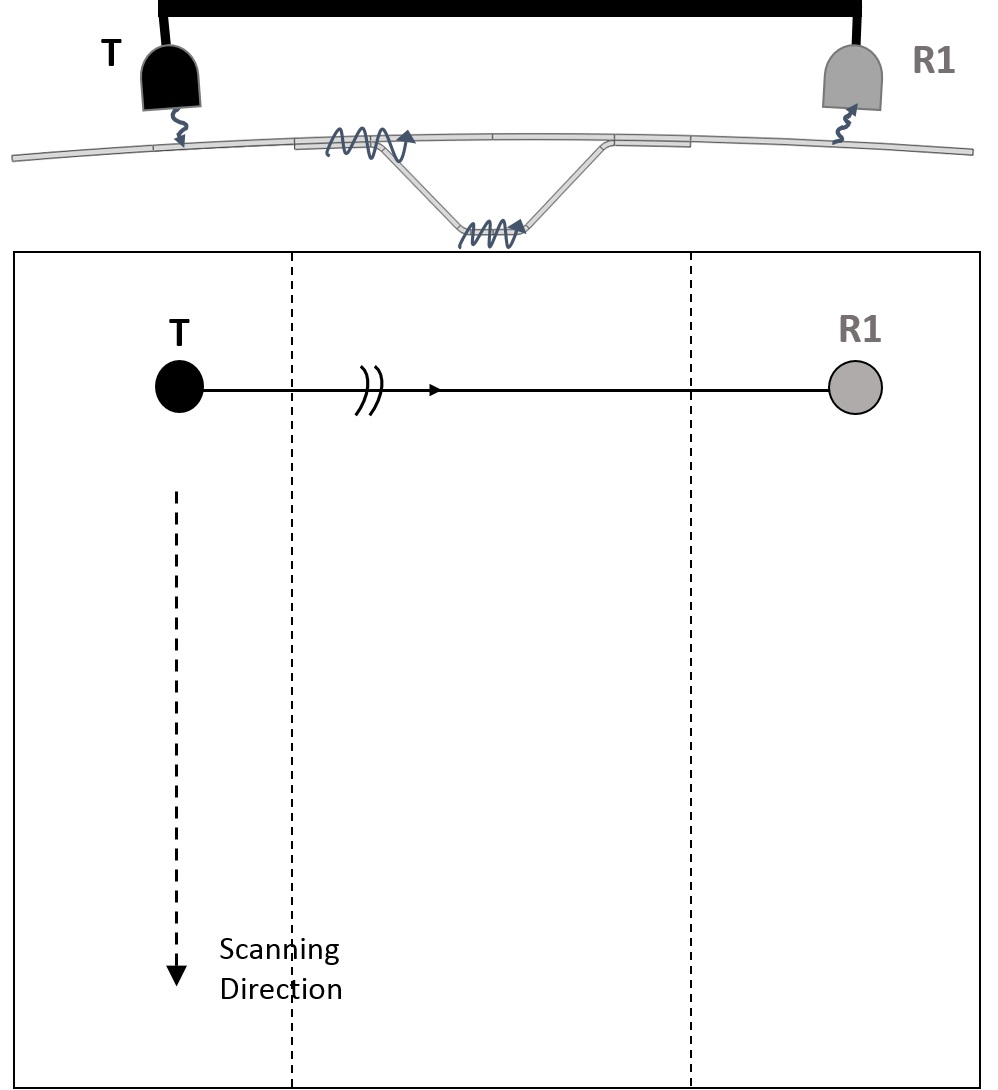

Supplement: Supplementary File 1 [file materials-10-00616-s001.zip › Figures_rev/Figure S4b_Line scanning approach for (b) non contact system.jpg]

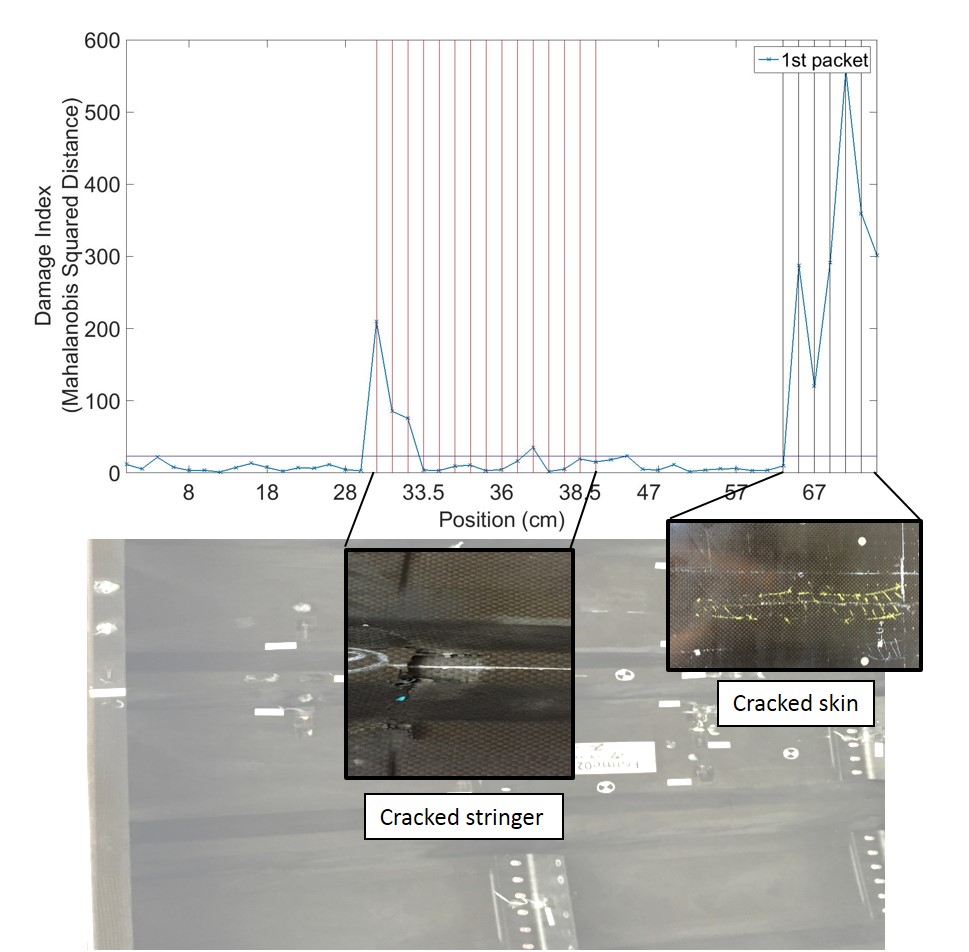

Supplement: Supplementary File 1 [file materials-10-00616-s001.zip › Figures_rev/Figure S5a_Representative results from contact UGW scans (a) Panel 1_rev.jpg]

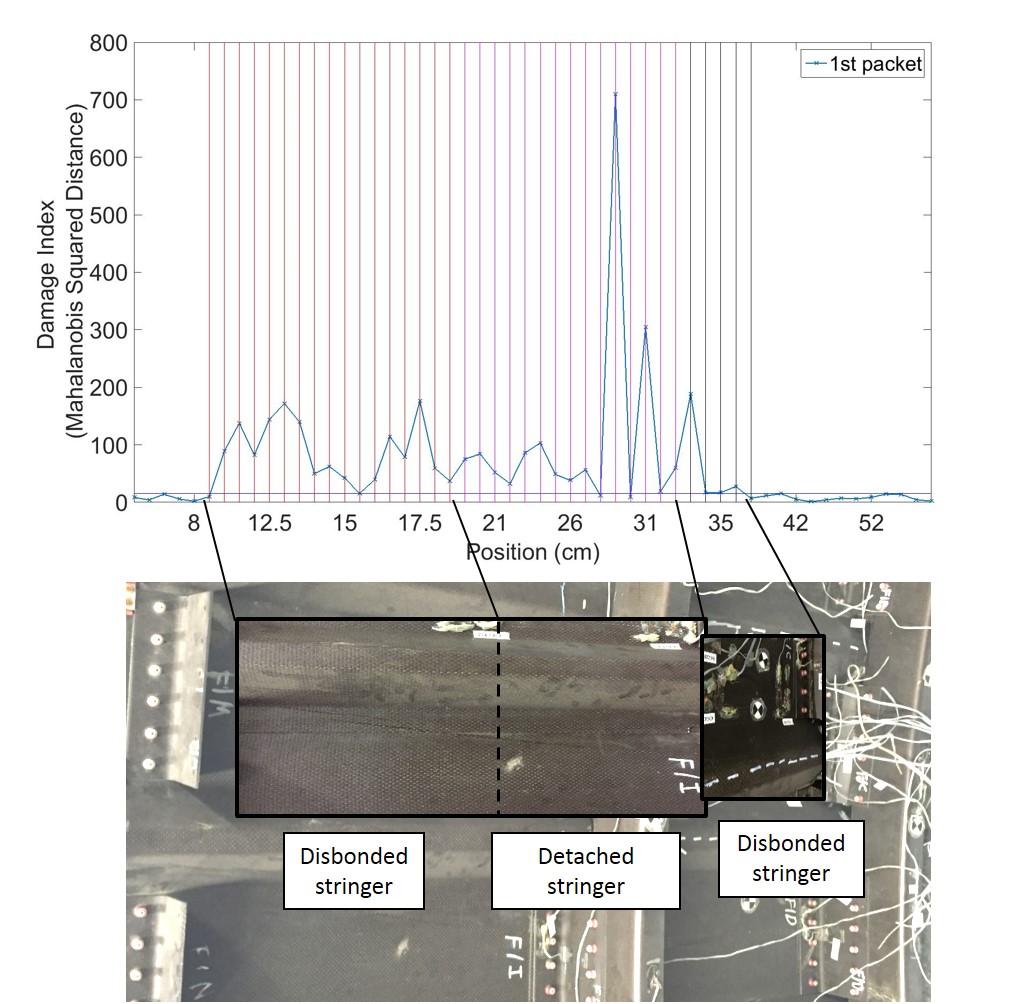

Supplement: Supplementary File 1 [file materials-10-00616-s001.zip › Figures_rev/Figure S5b_Representative results from contact UGW scans (b) Panel 2_rev.jpg]

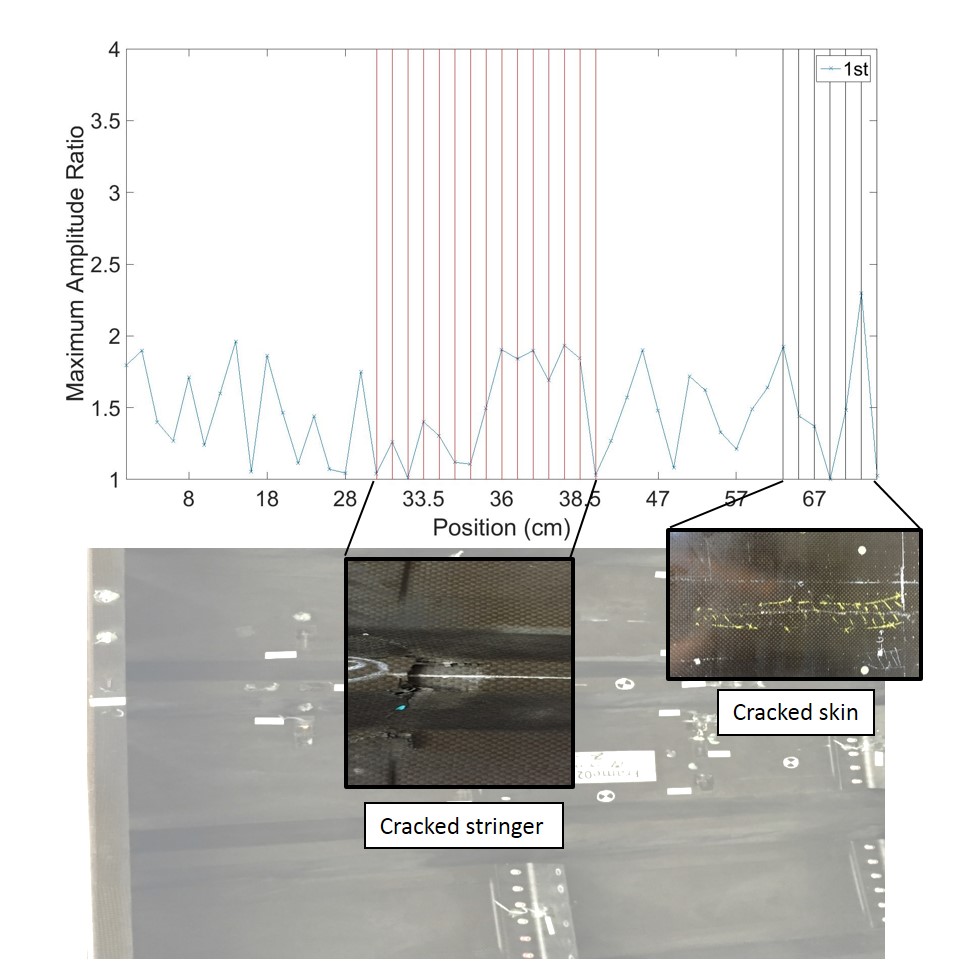

Supplement: Supplementary File 1 [file materials-10-00616-s001.zip › Figures_rev/Figure S6a_Amplitude Ratio from contact UGW scans (a) Panel 1.jpg]

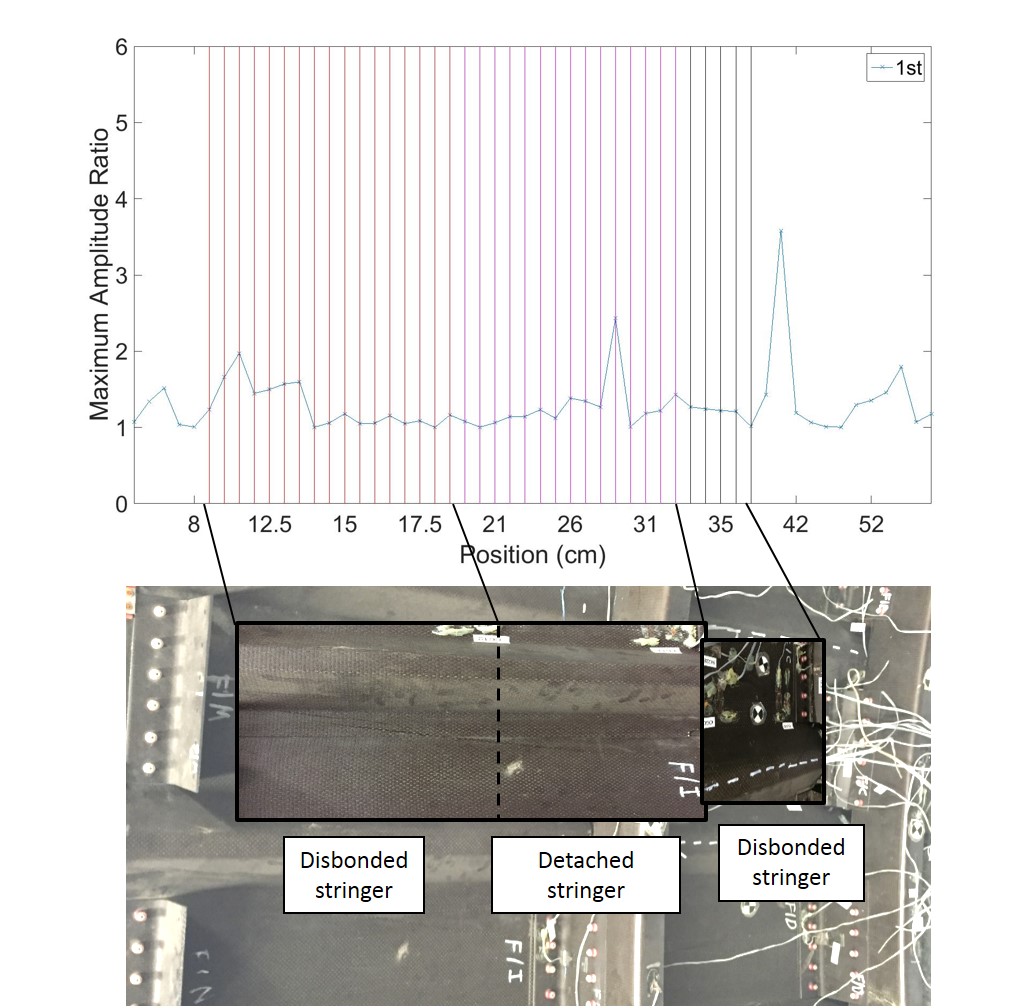

Supplement: Supplementary File 1 [file materials-10-00616-s001.zip › Figures_rev/Figure S6b_Amplitude Ratio from contact UGW scans (b) Panel 2.jpg]

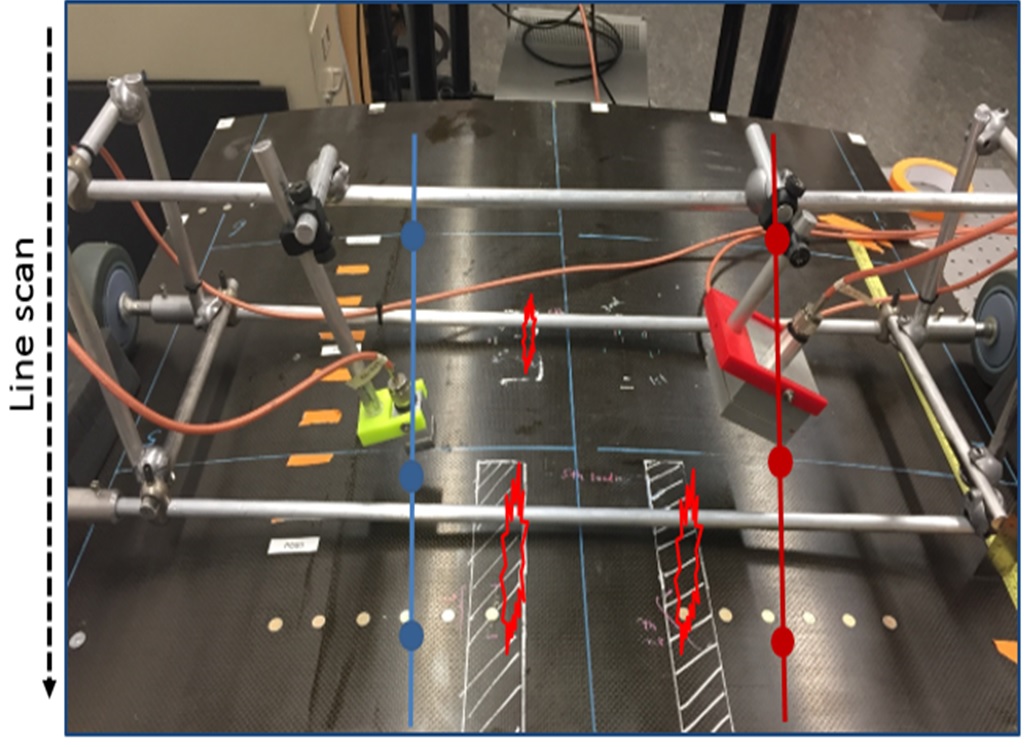

Supplement: Supplementary File 1 [file materials-10-00616-s001.zip › Figures_rev/Figure S7_Non contact air-coupled scanning prototype.jpg]

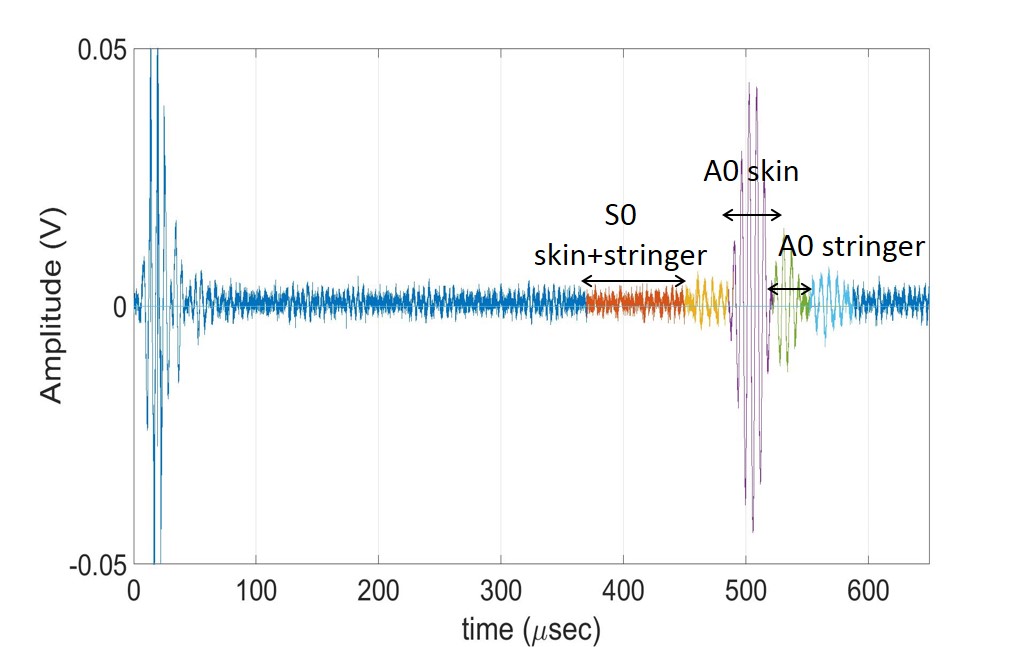

Supplement: Supplementary File 1 [file materials-10-00616-s001.zip › Figures_rev/Figure S8_Typical RF waveform measured by air-coupled.jpg]

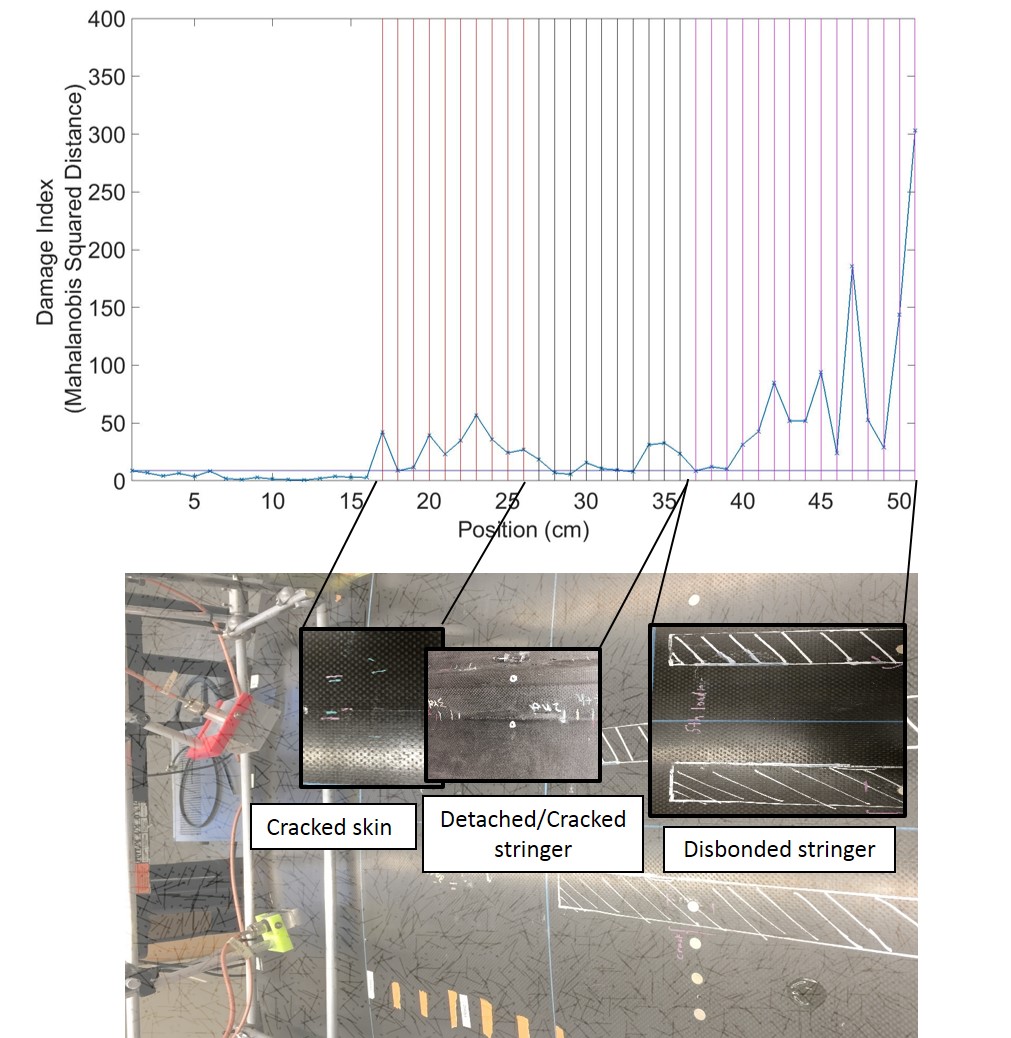

Supplement: Supplementary File 1 [file materials-10-00616-s001.zip › Figures_rev/Figure S9a_Representative results from noncontact (air-coupled) UGW scans of Panel 3 (a) skin modes only_rev.jpg]

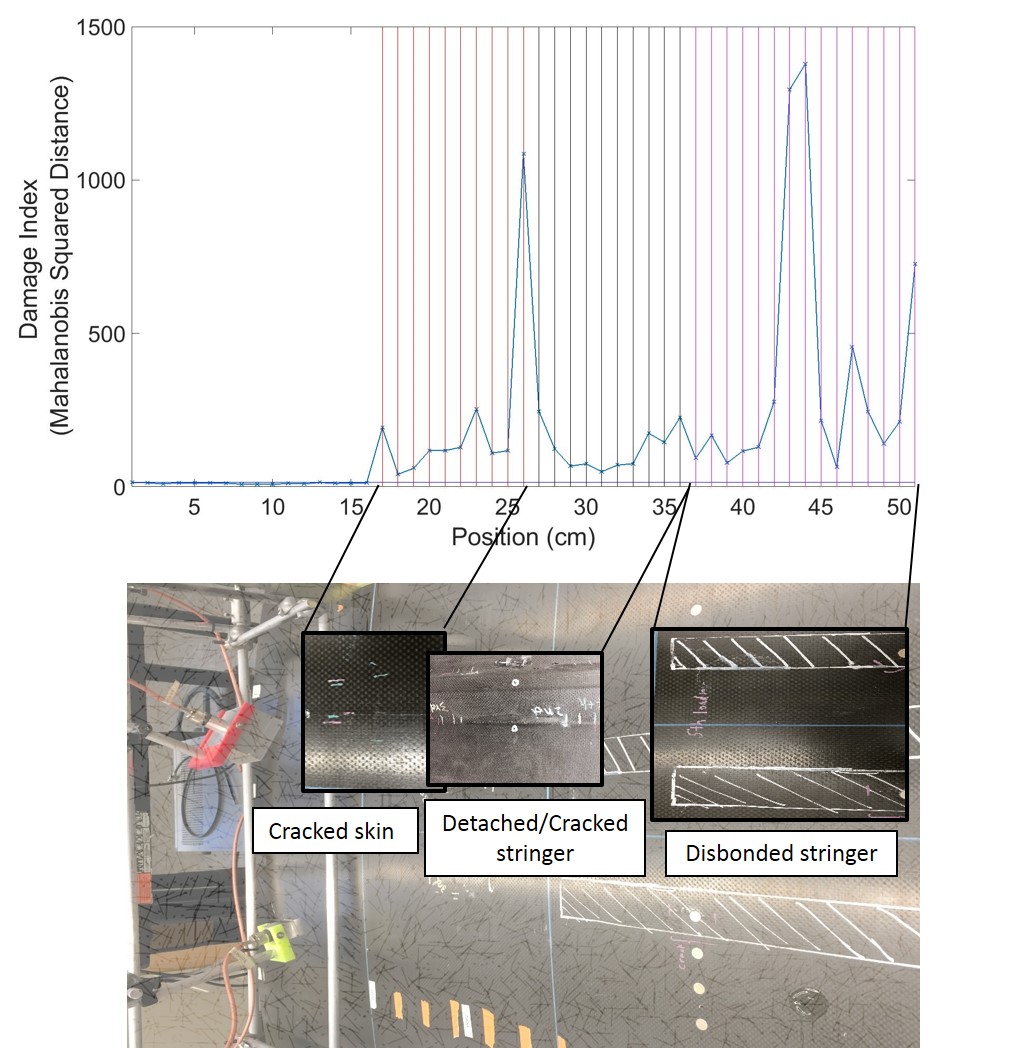

Supplement: Supplementary File 1 [file materials-10-00616-s001.zip › Figures_rev/Figure S9b_Representative results from noncontact (air-coupled) UGW scans of Panel 3 (b) skin modes plus stringer modes_rev.jpg]
